# Oral presentations at scientific meetings: some hints and tips

**DOI:** 10.1590/S1516-31802003000100010

**Published:** 2003-01-02

**Authors:** Eduardo Katchburian

It might perhaps be an exaggeration to say that scientific meetings are boring. Boring or not, we all have to attend, at least to show that we are alive and well. It is said that one week after a meeting nobody remembers much, apart from the conversation that went on over a cup of coffee or tea, or better, over a glass of wine or a tankard of beer. Others would say that they only remember the country, the town or the resort where the meeting took place! The reason for this is that most presentations tend to be extremely boring, simply because – as some cynics say – most scientists are a bore anyhow. Or perhaps, if one is in a generous mood, one might say that most scientists do not prepare their presentations well enough and fail to observe some elementary rules.

To start with, if you are not clear in your mind about what you wish to convey, i. e. your brain is in a fuzzy inextricable state of confusion, there is no way you can communicate anything because, really and truly, you have nothing to communicate. When we attend a lecture or talk, we like to sit comfortably and listen to a nice well-prepared story that can be followed as easily as a nursery rhyme. And we are right. Why should we make an effort to listen to a confused and inarticulate speech when we can, instead, daydream, spent a few minutes in the world of fantasy, or better still, fall asleep! If you cannot follow the story you will inevitably start daydreaming. Men will probably exercise their sexual fantasies while women will dream of going to a shopping center or supermarket!

I have attended a lot of scientific meetings in my lifetime, and I must confess that only a few were interesting enough to make a lingering impression in my mind.

Here, I have compiled a few hints and tips, which might be useful when preparing an oral presentation. Some colleagues will naturally disagree on some points. Giving a talk is a highly individual activity and we all have our own way of doing it.

1. Unless you are a big star (most of us are not), or you have a friend in the organizing committee, you will only be allowed 10 to 20 minutes for your talk. Remember that in well organized meetings when they tell you that you have 10 minutes, they mean 10 minutes. It is not considered polite to go over your time. You will most certainly be told to stop, a rather unpleasant situation.
2. In such a short time you will only be able to show a maximum of 15 to 20 highly selected images no matter what medium you use i.e. slides, overheads or data-show. One image per minute is usually what is necessary. Temptation to show all your wonderful images must be resisted. Remember, you are not giving a slide show: you are giving a talk!
3. Make sure you decide what story you want to tell beforehand. It may seem obvious but it is not! Re-assessing your results critically and without emotion will help you select what is worth telling. You will probably discover that a lot of stuff is not good enough for a presentation, sad as it may be. Remember, also, that a paper written for publication or already published is a poor basis for an oral presentation. You need a new script, not a short version of the paper, and new, purpose made, less complex illustrations.
4. Tell only one story. It is well documented that people hardly remember a lecture given by a Nobel laureate, let alone a 10-15 minutes talk by mere mortals like us. Never ever drift off from the main theme: it is fatal. People will start daydreaming, remember. You must capture the full attention of the audience at the very beginning otherwise it may never be secured. State objectives, concepts and ideas clearly. Eliminate irrelevant and distracting details.
5. Use clear, precise and simple English. Do not use more words than necessary. We know too well that it is easier to complicate things. You are a genius if you are able to make things look and sound simple. Avoid difficult words. You may sound pedantic or, worse still, you may sound as though you want to be pedantic! Some people think that long and complex sentences, somehow, miraculously, will enhance the importance of the subject and hence of your talk. In fact it is quite the opposite. It amazes me to see people coming out of a lecture saying that, although they did not understand a word the speaker said, they believed he was genius. According to Karl Popper you only understand something if you can explain it.
6. Write your text – I should say talk – indicating projection points and pauses clearly. Writing helps you think and it is indispensable for a clear and logical development of your ideas. In the process of writing you will discover inconsistencies and flaws in your reasoning. Most people who improvise usually prepare their improvisation carefully. Do not read your text while giving your talk. A speaker cannot hold the attention of an audience if he buries his head in a text. It is a talk, not a reading session, after all! But you must have it written down. In case you panic it is there, in front of you, thank God!
7. When projecting images containing text, do not read aloud the text on the slide. The audience can do that. Slides, overheads or data-show must be used to show evidence clearly and to the point. If you read the text on a slide, the audience doesn't know whether to pay attention to what you are saying, or to read the projected text and pay no attention to you. Thus, slides must not be used as *aides-mémoire*. Inadequate speakers who face the screen, i.e. turn their backs to the audience and read aloud the text on each slide, simply irritate the audience, and worse still, show that they could not be bothered to prepare their presentation. They must never be invited to give a talk again!
8. Do not deliver your presentation in total darkness. If possible try to have the lights on once or twice. People may wake up – if you are lucky! Remember, also, that you are competing with powerful daydreaming images.
9. Always look directly to your audience, i.e. never ever turn your back. Make eye contact but don't seek approval. Don't look up to the ceiling or down to the floor. The audience may think you are invoking the help of God…. Do not gesticulate too much, or walk up and down the aisles or along the rostrum. This advice is fine but unnecessary for Anglo-Saxons. If you are Italian, however, it maybe difficult to follow! Use the pointer to show something and don't keep moving it as you speak. Lasers pointers are more difficult to handle. They show more readily that you are terrified!!
10. Always inspect the lecture hall beforehand. Make sure you check projectors, microphones and lights. Data-show, in particular, is prone to last minute hiccups. Make sure you know who is doing the projection. If it is you, try the system beforehand.
11. Jokes, yes, jokes. Do not ever tell a joke unless you are a fantastic raconteur. Most people are not. It is safer to give a clear, sharp and interesting talk.
12. Make sure the audience can hear you. Obvious, isn't it?
13. Rehearse, rehearse, and rehearse again. Rehearse in front of friendly but critical friends. Time yourself precisely.

## VISUAL AIDS

It seems that the days of conventional slides and overheads are over. Most people have their digital images in CD-ROMs and will consequently use data-show. However the general principles for presentation remain virtually unchanged. Unfortunately, when misused or perhaps, I should say, abused, data-show can be a disaster.

1. Do not cram too much information in. Images are meant to show evidence as clearly as possible. They indicate points of anchorage or reference for the audience. You must select your images objectively and critically. Unfortunately, use of data-show has encouraged people to cram as much information as possible into a single image. So be ruthless with yourself and resist the temptation to overdo it.
2. Do not use too many colors. Use colors in a meaningful manner to help the audience, not to confuse it. Someone once said that slides/data-show are not birthday cakes. More often than not, plain black and white is more informative. I must confess, however, that in these days of media hype, it is difficult to convince people that simplicity is better than complexity. Some people love to show little images, words or sentences flying off the screen, right, left and center!
3. Use multiple projection when absolutely necessary and mainly to compare things. I have seen people use triple projection in a totally meaningless manner and I can only say that it was simply ridiculous!
4. So, here I go again: Refrain from using computer gimmicks, pies, blocks, data-show fireworks and other artifacts to impress your audience. Most people – usually the people that really matter – will be irritated and unimpressed. It is unfortunate that scientists from developing countries are particularly taken by extravagant and meaningless use of gimmickry and fireworks! And beware: there are clear signs on the horizon that the passion for technology is cooling off. Executives of large corporations are being told that they should rediscover the art of face-to-face communication rather than using frigid electronic technology. In the end, human nature will prevail.
5. Project your images in a lecture hall. Go to the back of the hall and check if you can see and read everything. Simply examining your images in your office can be deceiving.
6. Last but not least, remember that BEAUTY IS NO COVER FOR LACK OF CONTENT.

